# Mesenchymal stem cells empower T cells in the lymph nodes via MCP-1/PD-L1 axis

**DOI:** 10.1038/s41419-022-04822-9

**Published:** 2022-04-18

**Authors:** Yifan He, Yan Qu, Bowen Meng, Weiying Huang, Jianxia Tang, Runci Wang, Zetao Chen, Xiaoxing Kou, Songtao Shi

**Affiliations:** 1 Hospital of Stomatology, Guanghua School of Stomatology, Sun Yat-sen University, South China Center of Craniofacial Stem Cell Research, Guangdong Provincial Key Laboratory of Stomatology, 510055 Guangzhou, China; 2 Hunan Key Laboratory of Oral Health Research & Hunan Clinical Research Center of Oral Major Diseases and Oral Health, Xiangya School of Stomatology, Xiangya Stomatological Hospital, Central South University, 72 Xiangya Road, 410000 Changsha, Hunan China; 3 Shanghai Institute of Rheumatology/Department of rheumatology, Renji Hospital, Shanghai Jiaotong University School of Medicine, 200002 Shanghai, China; 4 Key Laboratory of Stem Cells and Tissue Engineering (Sun Yat-Sen University), Ministry of Education, 510080 Guangzhou, China

**Keywords:** Apoptosis, Mesenchymal stem cells, Immune cell death, T cells

## Abstract

Mesenchymal stem cells (MSCs) are a type of immunosuppressive stromal cell found in multiple tissues and organs. However, whether MSCs possess immunosupportive characteristics remains unclear. In this study, we showed that the lymph nodes contain immunosupportive MSCs. They produce and secrete a high level of MCP-1 to promote T-cell proliferation and differentiation, in contrast to bone marrow MSCs (BMMSCs), which repress T-cell activation. Unlike BMMSCs, lymph node MSCs (LNMSCs) fail to respond to activated T-cell-induced production of PD-L1 to induce T-cell apoptosis. Mechanistically, MCP-1 activates phospho-Erk to sustain T-cell proliferation and activation while it represses NF-κB/PD-L1 pathway to avoid induction of T-cell apoptosis. Interestingly, inflammatory lymph node-derived LNMSCs abolish their immunosupportive function due to reduction of MCP-1 expression. Finally, we show that systemic infusion of LNMSCs rescues immunosuppression in cytoxan (CTX)-treated mice. This study reveals a previously unrecognized mechanism underlying MSC-based immunoregulation using the MCP-1/PD-L1 axis to energize T cells and suggests a potential to use MSCs to treat immunosuppressive disorders.

## Introduction

The lymph nodes (LNs) are special lymphoid organs of the immune system and play a crucial role in the immune defense of the human body [1]. Over 500 LNs are strategically located at locations where they can be easily reached by immune cells traveling around the body, which makes the LNs the critical meeting points for the initiation of immune responses to antigens [2, 3]. LN functions include filtering pathogens, fighting infections and preventing cancer migration [1–3]. Disorders of the LN microenvironment may result in several immune diseases [4–6]. Therefore, further unveiling the role of this microenvironment is of great value.

Each LN is divided into the outer layer (cortex) and the inner layer (paracortex). The cortex is mostly populated by B-cell areas, and the paracortex contains the dendritic cells (DCs) and T cells [7, 8]. Antigens and DCs reach the LN through afferent lymphatic vessels; they then migrate deep into the LN to activate T cells. Macrophages are closely integrated with lymphatic endothelial cells to sample pathogens and antigens [7–9]. All these structures are maintained by a network of stromal cells, which can also actively influence immune responses.

The significance of the stroma in coordinating immune cell migration and positioning within LNs is well documented. Nevertheless, the detailed contributions of the stromal cells to immune cell homeostasis and adaptive immunity are largely unknown. Among the various subsets of LN stromal cells, follicular dendritic cells densely populate in the B-cell areas and produce B-cell survival factors such as APRIL and BAFF [10, 11]. Fibroblastic reticular cells (FRCs) are found in the T-cell areas of the LN paracortex and contribute in recruitment and survival of T cells [12–14]. FRCs are also known to limit antibody responses and plasma cell survival via monocyte accumulation and reactive oxygen species [15].

In recent years, it has been gradually recognized that LN mesenchymal stem cells (MSCs) coexist with the active immune cells [16]. Unlike primary lymphoid organs such as bone marrow, secondary lymphoid organs are important places where immune cells settle, activate, and proliferate, and where immune responses occur. T cells represent most of lymph cells in the LNs and play a critical role in cellular immune responses and host defense [17]. A large number of physiological processes like T-cell proliferation, differentiation, activation and apoptosis take place continuously in the LNs to maintain the local immune homeostasis and sustain the function of cellular immune responses [18].

Since MSCs were found to exhibit strong immunoregulatory function [19], a substantial body of literature has investigated the interactions between MSCs and immune cells. It has been generally accepted that MSCs exert anti-inflammatory effects on several major immune cells, such as T and B lymphocytes, natural killer cells and dendritic cells, by inhibiting their proliferation and activation while promoting their apoptosis [20–22]. MSCs derived from different sources, such as bone marrow, umbilical cord, adipose tissue, and dental pulp, have been employed in the treatment of a number of refractory autoimmune diseases with promising results [23–25]. So far there is no convincing evidence to show that MSCs possess immunosupportive function.

In this study, we found that lymph node MSCs (LNMSCs) show a distinct immunosupportive function, rather than an immunosuppressive function like that of bone marrow MSCs (BMMSCs). LNMSCs empower T cells by promoting their proliferation and suppressing their apoptosis. Nevertheless, these functions can be reversed under inflammatory conditions via regulation of the MCP-1/PD-L1 axis.

## Results

### LNMSCs energize T cells

We found that LNMSCs show classical MSC morphology (Supplementary Fig. S1A) coupled with the capacity for self-renewal, as assessed by proliferation rate and colony-forming units (Supplementary Fig. S1B, C). Flow cytometric analysis confirmed that LNMSCs were positive for the MSC surface molecules such as CD44, CD73, CD90, Sca-1, and CD105, but were negative for the hematological markers such as CD45 and CD34 (Supplementary Fig. S1D). LNMSCs also showed the capacity for multipotent differentiation into osteogenic, adipogenic and chondrogenic lineages (Supplementary Fig. S1E-I).

**Fig. 1:**
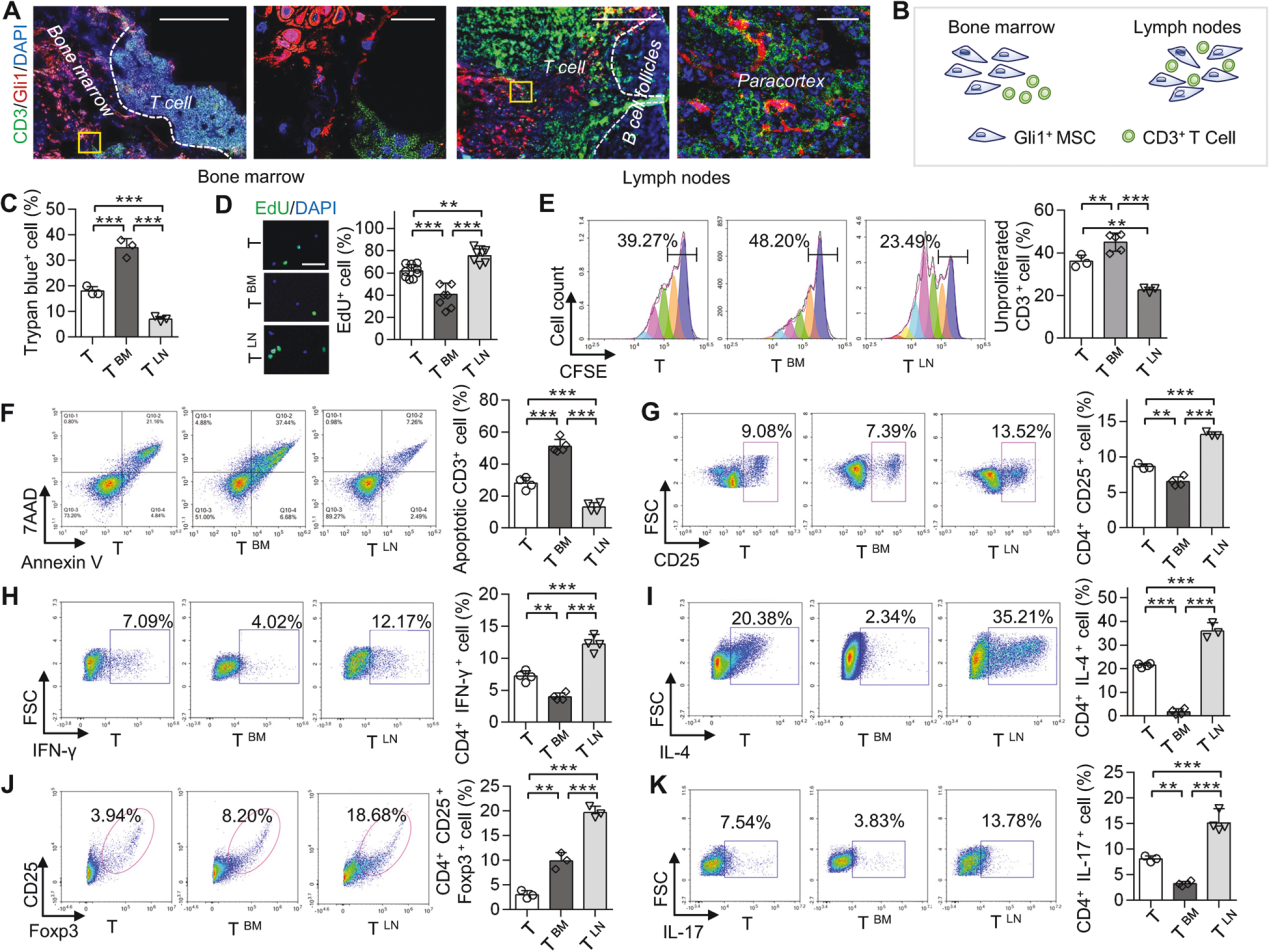
Immunosupportive effect of LNMSCs. **A** The distribution of Gli1-positive MSCs in the lymph nodes and bone marrow of *Gli1-Cre*^*ERT2*^*; R26*^*tdTomato*^ mice. Scale bars = 200 μm for lower magnification and 20 μm for higher magnification. **B** Schematic figure of the distribution of Gli1-positive MSCs in mouse lymph nodes and bone marrow. **C** Live-cell counting of trypan blue-positive T cells after co-culture with LNMSCs and BMMSCs. *n* = 3. **D**, **E** CFSE analysis and EdU staining showed that LNMSCs promote activated T-cell proliferation. Scale bar = 50 μm. *n* = 7–9 (**D**), *n* = 3–5 (**E**). **F** Flow cytometry analysis showed the anti-apoptotic effect of LNMSCs on activated T cells. *n* = 4–5. **G** Flow cytometry analysis showed that LNMSCs stimulate CD4^+^ T-cell activation. *n* = 3-4. **H**–**K** Flow cytometry analysis showed that LNMSCs and BMMSCs regulated T-cell differentiation. *n* = 4 (H), *n* = 3–4 (**I**), *n* = 3 (**J**), *n* = 3–4 (**K**). *P < 0.05, **P < 0.01, ***P < 0.001.

*Gli1-Cre*^*ERT2*^ mice were crossed with *R26*^*tdTomato*^ mice to examine the distribution pattern of LNMSCs. We used immunofluorescence staining to show that Gli-1-positive cells were specifically distributed around or at the edge of the T-cell aggregation area in the LNs, when compared with the bone marrow (Fig. 1A, B). To assess the in vitro immunomodulatory capacity of LNMSCs, we co-cultured LNMSCs or BMMSCs with activated T cells for 1 day, which showed a significant decrease of trypan blue-positive T cells in the LNMSC group compared to the BMMSC control group (Fig. 1C). Flow cytometry analysis showed that LNMSCs were capable of promoting T-cell proliferation and activation while BMMSCs appeared to have the opposite effects, blocking T-cell proliferation and activation (Fig. 1E, G). These results were further confirmed by EdU staining (Fig. 1D). After coculturing LNMSCs with activated T cells for 4 days, we observed that LNMSCs could promote T-cell proliferation from day 1 to 4, as assessed by CFSE analysis (Supplementary Fig. S2A). Moreover, we found that LNMSCs were able to induce T-cell differentiation (Fig. 1H–K) and suppress T-cell apoptosis (Fig. 1F). In contrast, BMMSCs promoted T-cell apoptosis (Fig. 1F). When we co-cultured LNMSCs with activated total T cells, CD4^+^ T cells, CD8^+^ T cells and B cells for 5 days, we found that LNMSCs significantly suppressed the apoptosis of all T cells, as well as B cells and reduced the CD4/CD8 ratio (Supplementary Fig. S2B–G). These results indicated that LNMSCs have immunosupportive effects on activated T cells by supporting their proliferation and activation but suppressing their apoptosis. When compared to bone marrow, lung and spleen, LN also showed superior capacities to promote CD3^+^ T-cell proliferation, activation, and apoptotic resistance (Supplementary Fig. S3A–C).

### MCP-1 is required to maintain immunosupportive effect of LNMSCs

**Fig. 2:**
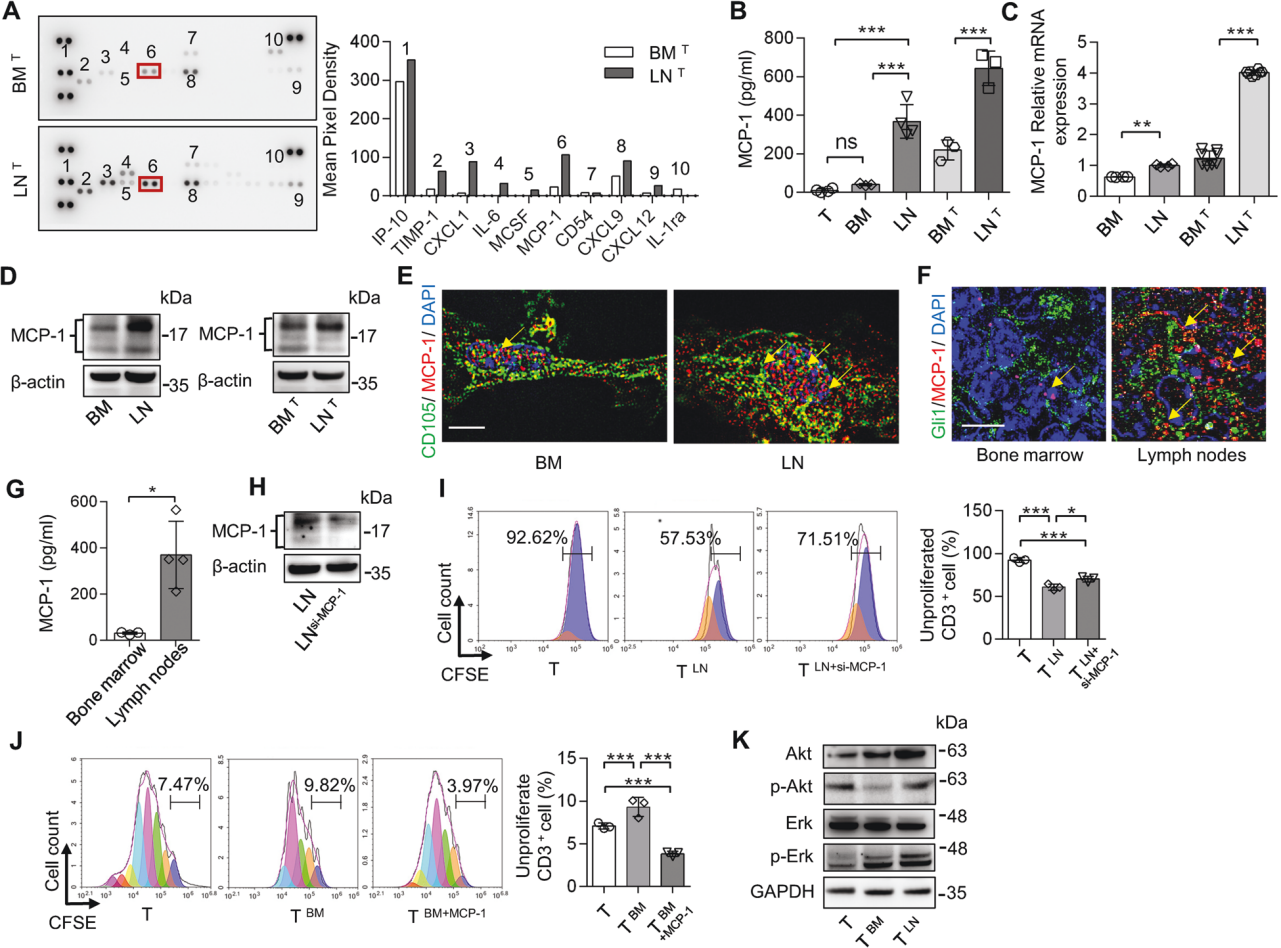
LNMSCs secrete MCP-1 to promote T-cell proliferation. **A** Cytokine array analysis showed that supernatants of T cells co-cultured with LNMSCs contained a higher level of MCP-1 than those co-cultured with BMMSCs. **B**–**D** ELISA, real-time qPCR and Western blotting results showed that LMNSCs produced and secreted a higher level of MCP-1 than BMMSCs before and after co-cultured with activated T cells. *n* = 3–4 (**B**), *n* = 4–8 (**C**). **E** LNMSCs expressed an elevated level of MCP-1 compared to that of BMMSCs in vitro. Scale bar = 5 μm. **F**, **G** The lymph nodes contain a higher level of MCP-1 than bone marrow. Scale bar = 5 μm. *n* = 3–4 (**G**). **H**, **I** MCP-1 siRNA treatment significantly reduced the capacity of LNMSCs to induce T-cell proliferation. *n* = 3 (**I**). **J** MCP-1 treatment reversed the ability of BMMSCs to promote T-cell proliferation. *n* = 3. **K** Western blotting showed LNMSCs promoted T-cell proliferation via Erk signaling pathway and BMMSCs suppressed T-cell proliferation via Akt signaling pathway. *P < 0.05, **P < 0.01, ***P < 0.001.

To explore the underlying mechanism by which LNMSCs promote T-cell function, we assayed the supernatants of LNMSCs or BMMSCs after co-culture with activated T cells. Cytokine array analysis showed that the co-culture supernatant of LNMSCs contained a higher level of MCP-1 than that of BMMSCs (Fig. 2A). ELISA, real-time qPCR, immunofluorescence staining, and Western blotting analysis further confirmed that LNMSCs produced and secreted higher levels of MCP-1 than BMMSCs (Fig. 2B–E). After co-culture with activated T cells, the concentration of MCP-1 increased significantly in LNMSCs, as well as in the co-culture supernatant (Fig. 2B–D). Next, we showed that LNs contain a significantly elevated level of MCP-1 relative to the bone marrow in *Gli1-Cre*^*ERT2*^*; R26*^*tdTomato*^ mice, assessed by ELISA and immunofluorescence staining (Fig. 2F, G).

To further determine the role of MCP-1 in the immunosupportive function of LNMSCs, we used MCP-1 siRNA to knock down MCP-1 expression in LNMSCs and found that MCP-1 siRNA knockdown LNMSCs showed a significantly reduced capacity to promote T-cell proliferation (Fig. 2I). Western blotting results confirmed the decreased expression level of MCP-1 in siRNA-treated LNMSCs compared to the control group (Fig. 2H). Moreover, MCP-1 treatment increased BMMSC-mediated T-cell proliferation (Fig. 2J). Based on the above findings, we examined how MCP-1 regulates T-cell proliferation. It is known that MCP-1 binding to CCR2 in monocytes can elicit the activation of the extracellular-signal regulated kinase (Erk) and phosphatidylinositol 3-kinase (PI3K) pathways, which govern monocyte activation and proliferation [26]. We found that LNMSCs can induce Erk phosphorylation in T cells, as assessed by Western blotting analysis. In contrast, the phosphorylation of Akt was inhibited only in the BMMSC group (Fig. 2K). These results suggest that LNMSCs use MCP-1 to promote activated T cells via the Erk signaling pathway.

### Proteomics analysis of LNMSCs

**Fig. 3:**
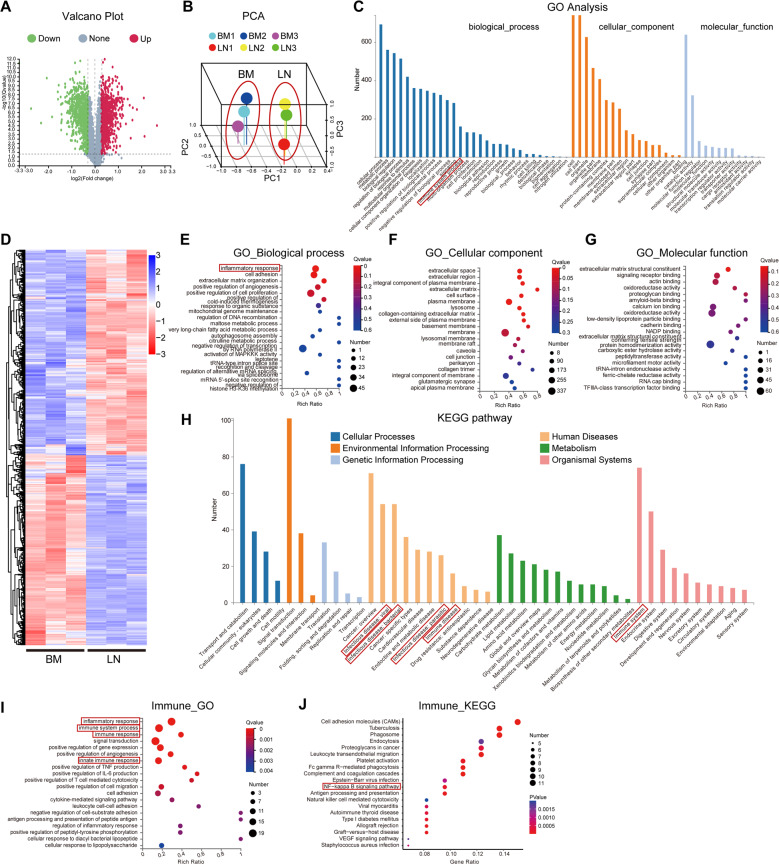
iTRAQ quantitative proteomic comparison between LNMSCs and BMMSCs after co-culture with activated T cells. **A** Volcano plot showing the differentially expressed proteins (DEPs). **B** Principal component analysis showed obvious separation of samples from different groups. **C** GO classified annotation of differential expressed proteins. **D** Cluster heatmap of gene expression profiles. Blue indicates downregulation, red indicates up-regulation. **E**–**G** GO enrichment analysis of DEPs on biological process (**E**), cellular component (**F**), and molecular function (**G**) levels. **H** KEGG classified annotation of DEPs. **I** GO enrichment analysis of the immune process-related proteins of DEPs on biological process level. **J** KEGG analysis of the immune process-related proteins of DEPs. *n* = 3.

**Fig. 4:**
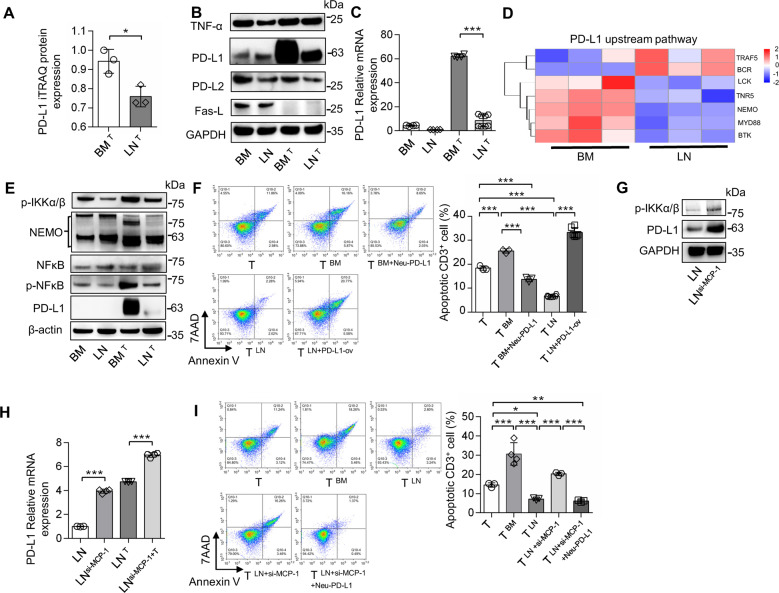
LNMSCs suppress activated T-cell apoptosis via MCP-1/PD-L1 axis. **A**–**C** The elevated expression of PD-L1 was significantly suppressed in LNMSCs after co-culture with T cells. *n* = 3 (**A**), *n* = 4–8 (**C**). **D**, **E** BMMSCs promote T-cell apoptosis via NF-κB/PD-L1 signaling pathway. **F** Blocking PD-L1 in BMMSCs abolished their function to promote T-cell apoptosis. Overexpression of PD-L1 in LNMSCs rescued their capacity to promote T-cell apoptosis. *n* = 3–6. **G**, **H** Western blotting showed significantly elevated expression of PD-L1 in LNMSCs after treatment with MCP-1 siRNA. The inhibition of PD-L1 by MCP-1 in LNMSCs is associated with NF-κB/PD-L1 signaling pathway. *n* = 3–4 (**H**). **I** Downregulation of the expression of MCP-1 in LNMSCs markedly increased the T-cell apoptotic rate when co-cultured with the siRNA-treated LNMSCs, while the blocking of PD-L1 reversed the T-cell apoptotic rate increase caused by the usage of MCP-1 siRNA. *n* = 3-4. *P < 0.05, **P < 0.01, ***P < 0.001.

To examine specific proteomic features of LNMSCs and BMMSCs in immune regulation, we prepared LNMSC and BMMSC proteins after co-cultured with T cells and performed LC-MS/MS analysis. The isobaric tags for relative and absolute quantitation (iTRAQ) showed a total of 6,696 proteins with 1% FDR (Table 1), while 2150 proteins were differentially expressed between LNMSCs and BMMSCs (fold-change > 1.2, *Q*-value < 0.05) (Fig. 3D), which included 1117 significantly upregulated proteins and 1033 significantly downregulated proteins in LNMSCs (Fig. 3A). Principal component analysis showed an obvious separation of samples from LNMSCs and BMMSCs (Fig. 3B), indicating their distinct protein expression pattern. We next focused on the differentially expressed proteins (DEPs) and conducted a functional analysis. Gene ontology (GO) classified annotation showed that the DEPs were annotated to multiple terms within the three categories “Biological process”, “Cellular component”, and “Molecular function”, suggesting load of various functional proteins in LNMSCs and BMMSCs (Fig. 3C). GO biological process enrichment analysis unveiled enrichment of inflammatory response, cell adhesion and extracellular matrix organization in biological process (Fig. 3E), indicating the potential regulatory function of DEPs. GO cellular component enrichment analysis showed the most enriched 6 terms were related to extracellular space, plasma membrane, etc. (Fig. 3F), while no term was considered significantly enriched (*Q*-value < 0.05) in GO molecular function enrichment analysis (Fig. 3G). Kyoto Encyclopedia of Genes and Genomes (KEGG) pathway annotation results showed that these DEPs have multiple functional characteristics, which are associated with “infections disease: viral”, “infections disease: bacterial”, “infections disease: parasitic”, “immune disease” within the “human diseases” domain, and “immune system” within the “organismal systems” domain (Fig. 3H). Notably, the immune system categories, containing 74 DEPs, were one of the most enriched categories, suggesting that LNMSCs and BMMSCs may possess different immune regulatory functions. Further GO biological process enrichment analysis of DEPs in the immune system category indicated that these differences mainly pertain to the inflammatory response, immune system process, immune response and innate immune response (Fig. 3I). These findings suggest that DEPs are consisted of a set of functional proteins that are highly related to immunity and inflammation. Furthermore, KEGG analysis of DEPs in the immune system category indicated that the NF-κB signaling pathway was one of major significantly enriched pathway (Fig. 3J). NF-κB signaling was considered as one of the most important upstream pathways to promote PD-L1 expression [27]. PD-L1 expression was significantly downregulated in LNMSCs as identified by iTRAQ quantification (Fig. 4A). The cluster heatmap showed proteins associated with NF-κB signaling pathway significantly reduced in LNMSCs (Fig. 4D), indicating the NF-κB-PD-L1 signaling pathway was suppressed in LNMSCs when compared to BMMSCs. Moreover, the cluster heatmap of proteins associated with JAK family showed that JAK-1, which activates downstream STAT pathways, thereby promoting MCP-1 expression, was markedly increased in LNMSCs compared to BMMSCs (Supplementary Fig. S4A). Real-time qPCR results further confirmed that LNMSCs expressed a higher level of JAK-1 than BMMSCs (Supplementary Fig. S4B). These results implied the underlying mechanisms of LNMSCs regulated T cells: LNMSCs expressed increased level of JAK-1 and decreased level of PD-L1 and its upstream NF-κB signaling pathway.

**Table 1:** Protein identification overview of iTRAQ quantification.

Species	Total spectra	Spectra	Unique spectra	Peptide	Unique peptide	Protein
Mus_musculus	852,299	89,548	83,704	37,538	36,423	6696

### LNMSCs suppress activated T-cell apoptosis via MCP-1/PD-L1 axis

We next determined how LNMSCs regulate T-cell apoptosis. The iTRAQ quantification, Western blotting and RT-qPCR results showed that the elevated expression of PD-L1 in BMMSCs after co-culture with T cells was significantly suppressed in the LNMSC group (Fig. 4A–C and Supplementary Fig. S5A). Also, PD-L1 neutralizing antibody treatment markedly reduced BMMSC-mediated promotion of T-cell apoptosis. Overexpression of PD-L1 in LNMSCs promoted their capacity to induce T-cell apoptosis (Fig. 4F). KEGG analysis and the heatmap of iTRAQ quantification proteomics showed a significantly reduced expression of proteins associated with NF-κB signaling pathway in the LNMSCs compared to the BMMSC group (Figs. 3J and 4D). Furthermore, Western blotting analysis showed that the expression levels of p-IKKα/β, NEMO, NF-κB p65, p-NF-κB p65, and PD-L1 increased in BMMSCs, but not in LNMSCs (Fig. 4E). Flow analysis showed MCP-1 siRNA treatment markedly increased the rate of T-cell apoptosis when co-cultured with the siRNA-treated LNMSCs (Fig. 4I), which was associated with the elevated expression of PD-L1 in LNMSCs after treatment with MCP-1 siRNA (Fig. 4G, H). MCP-1 siRNA treatment elevated the expression of PD-L1 in LNMSCs via NF-κB signaling pathway activation (Fig. 4G). We also found LNMSCs lost the capacity to increase T-cell apoptosis after using MCP-1 siRNA treatment in the PD-L1 neutralizing antibody treatment condition (Fig. 4I). Taken together, these data suggest that MCP-1 inhibits PD-L1 expression after co-culture with T cells via the NF-κB pathway.

### Inflammatory microenvironment diminishes LNMSCs’ immunosupportive effect

**Fig. 5:**
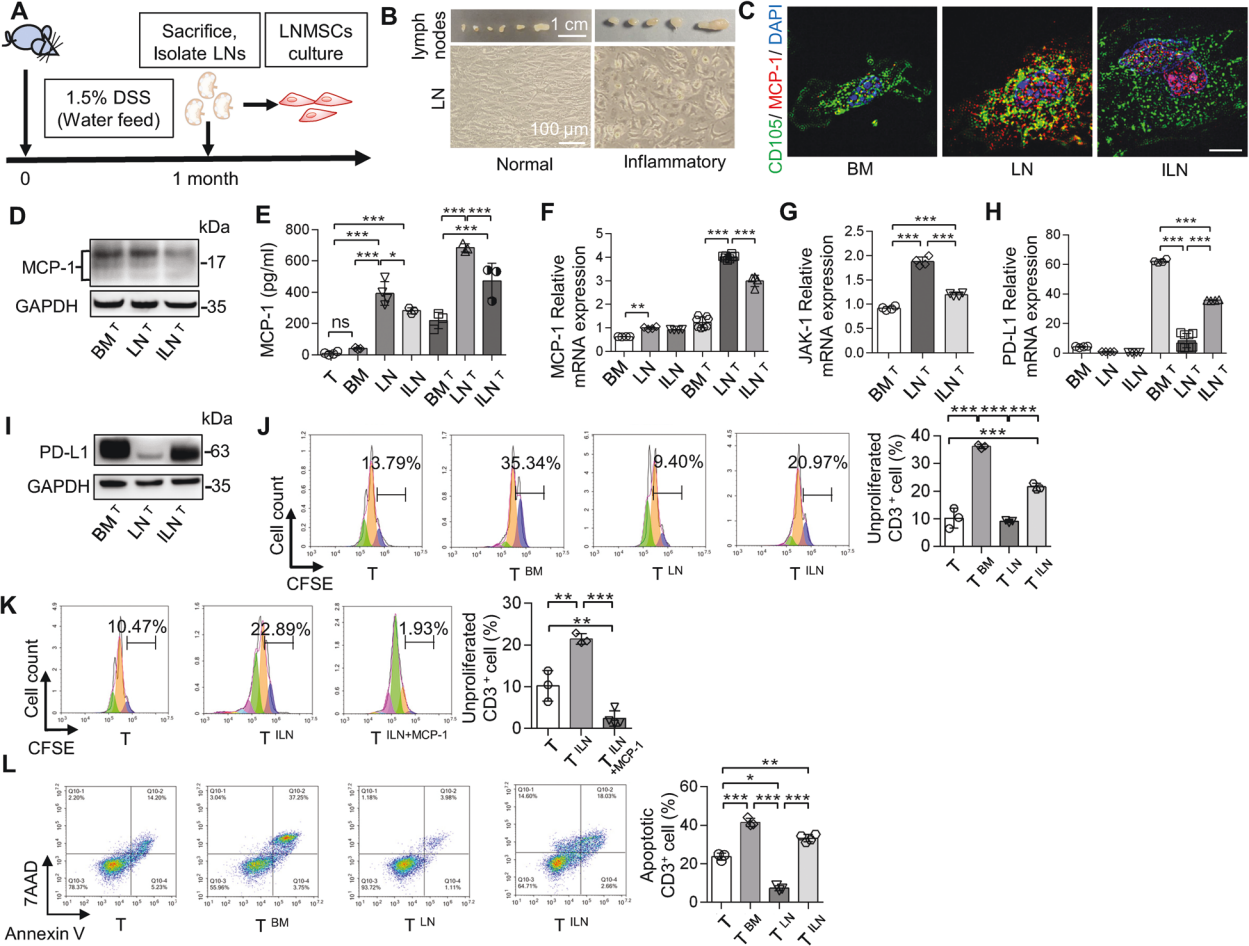
Inflammatory LNMSCs (I-LNMSCs) lose immunosupportive function. **A** I-LNMSCs were derived from the lymph nodes of DSS-induced inflammatory mice. **B** Morphology of inflammatory lymph nodes and I-LNMSCs. Scale bar = 1 cm and 100 μm. **C**–**F** Western blotting, immunofluorescence staining, real-time qPCR and ELISA results showed that I-LMNSCs produced and secreted less MCP-1 than normal LNMSCs. Scale bar = 5 μm. *n* = 3–4 (**E**), *n* = 4–8 (**F**). **G** I-LNMSCs expressed a lower level of JAK-1 than normal LNMSCs. *n* = 4. **H**, **I** I-LNMSCs expressed higher levels of PD-L1 than normal LNMSCs. *n* = 4–8 (**H**). **J**, **K** I-LNMSCs lost the ability to promote T-cell proliferation. MCP-1 protein treatment significantly recovered this capacity. *n* = 3 (**J**), *n* = 3–4 (**K**). **L** I-LNMSCs significantly increased T-cell apoptosis than normal LNMSCs. *n* = 3–4. *P < 0.05, **P < 0.01, ***P < 0.001.

Dextran sulfate sodium (DSS)-induced experimental chronic colitis has been widely used as a model of inflammation in mice, which display symptoms including swollen LNs. The immunoregulatory capacity of inflammatory LN-derived MSCs (I-LNMSCs) were evaluated in DSS-induced colitis mice (Fig. 5A, B, J). Western blotting, immunofluorescence staining, real-time qPCR and ELISA analysis showed that I-LMNSCs produced and secreted less MCP-1 than normal LNMSCs from control mice (Fig. 5C–F). Meanwhile, significantly lower mRNA expression of JAK-1 was observed in I-LNMSCs than normal LNMSCs (Fig. 5G). I-LNMSCs expressed much higher levels of PD-L1 than normal LNMSCs, as assessed by Western blotting and real-time qPCR (Fig. 5H, I). I-LNMSCs lost the function to promote T-cell proliferation, which could be recovered by adding additional MCP-1 (Fig. 5J, K). In addition, I-LNMSCs were able to induce T-cell apoptosis (Fig. 5L). These data suggest that LNMSCs lose the function to empower T cells under inflammatory conditions, which are associated with the low expression level of MCP-1 in I-LNMSCs.

### LNMSCs rescue CTX-induced immunosuppressed phenotypes

**Fig. 6:**
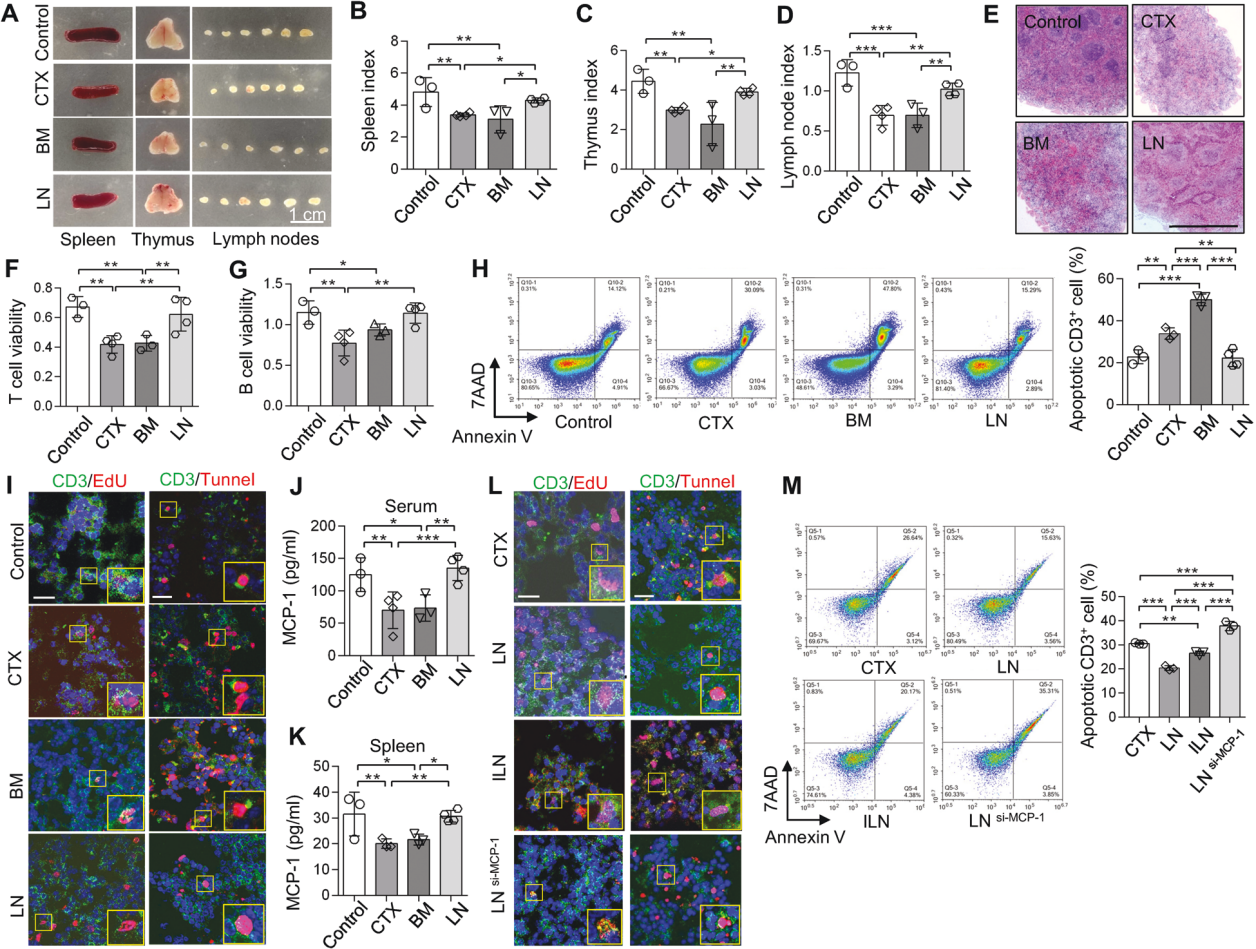
LNMSCs rescue immunosuppressed phenotypes in CTX-induced mice. **A** The morphology of immune organs (spleen, thymus and lymph nodes) decreased in CTX-induced immunosuppressed mice, and recovered after treatment with LNMSCs. Scale bar = 1 cm. **B**–**D** The immune indices of the spleen, thymus and lymph nodes decreased in CTX-induced immunosuppressed mice, and recovered after treatment with LNMSCs. *n* = 3–4 (**B**–**D**). **E** The pathological changes of spleens in CTX-induced mice. Scale bar = 1 mm. **F**, **G** CCK8 assay showed the viability of T cells and B cells of spleens recovered after treatment with LNMSCs. *n* = 3–4 (**F**, **G**). **H** The apoptotic rate of CD3^+^ T cells in the spleen decreased after treatment with LNMSCs and increased after treatment with BMMSCs. *n* = 3–4. **I** Immunofluorescence staining showed LNMSCs promoted the proliferation of T cells in spleens, while BMMSCs exhibited opposite effects. LNMSCs suppressed the death of T cells in spleens, while BMSCs induced it. Scale bar = 20 μm. **J**, **K** ELISA analysis showed MCP-1 significantly increased in serum and spleens of LNMSC-treated CTX-induced immunosuppressed mice. *n* = 3-4 (**J**, **K**). **L**, **M** Immunofluorescence staining and flow analysis showed I-LNMSCs and LNMSCs pre-treated with MCP-1 siRNA increased T-cell apoptosis and failed to promote T-cell proliferation in spleens of CTX-induced mice. Scale bar = 20 μm. *n* = 3. *P < 0.05, **P < 0.01, ***P < 0.001.

CTX-induced immunosuppressed mice show significant lymphoid organ atrophy, lymphopenia and pancytopenia. To evaluate therapeutic effect of LNMSCs, the morphological changes in the spleen, thymus and LNs were examined in CTX-treated immunosuppressed mice. The gross morphology and immune organ indices showed that infusion of LNMSCs markedly reversed the lymphoid organ atrophy in CTX-treated mice compared to the BMMSC-treated group (Fig. 6A–D). Histopathological staining suggested that LNMSCs partly rescued lymphopenia in the spleen, suggesting that LNMSCs protect the immune organs against CTX-induced impairment (Fig. 6E). To evaluate the protective effects of LNMSCs on the pancytopenia induced by CTX, the erythrocytes and platelets from the peripheral blood of CTX-treated mice were examined. Hematologic analysis showed that LNMSCs rescued the number of erythrocytes and platelets, suggesting that LNMSCs can restore the myelosuppression induced by CTX treatment (Supplementary Fig. S6A, B). ELISA results showed that CTX injection caused significant reductions in the levels of TNF-α, IFN-γ and IL-6. LNMSC treatment improved the reduced levels of TNF-α, IFN-γ and IL-6, while BMMSC treatment only improved the levels of TNF-α and IFN-γ (Supplementary Fig. S6C–E).

We next examined the mechanism of the LNMSC-mediated therapeutic effect in CTX-induced immunosuppressed mice. CCK8 assays showed LNMSCs significantly increased the viability of T cells and B cells of immune organs including the spleen and LNs of CTX-treated mice when compared to the BMMSC treatment group (Fig. 6F, G and Supplementary Fig S6F, G). EdU staining further confirmed that LNMSCs induced T-cell proliferation in the spleen when compared to BMMSCs (Fig. 6I). Meanwhile, flow analysis and immunofluorescence staining indicated that BMMSCs promoted T-cell apoptosis in the spleen while LNMSCs inhibited it (Fig. 6H, I). MCP-1 levels markedly increased in the serum and spleen of the LNMSC treatment group compared to the BMMSC group, as assessed by ELISA (Fig. 6J, K). In contrast, I-LNMSCs or LNMSCs pre-treated with MCP-1 siRNA failed to induce T-cell proliferation or suppress T-cell apoptosis in the spleen of CTX-treated mice (Fig. 6L, M). These results suggest that LNMSCs can ameliorate the immunosuppression symptoms of CTX-induced mice by secreting MCP-1 to promote T-cell viability and proliferation, as well as suppress T-cell apoptosis.

## Discussion

Previous studies showed that the activation of T cells in the LNs is a key initiating event in immune responses [28–30]. Our experimental results consistently showed that T cells derived from the LNs maintain a more active status compared to those from the lung, bone marrow and spleen. LNMSCs, a major component of the LN microenvironment, are capable of empowering T cells to increase their proliferation, differentiation, activation, and survival capacities.

Our study showed that the LNs contain tissue-specific MSCs with unique characteristics. LNMSCs are similar to BMMSCs in terms of their expression of surface markers CD105, CD90, CD44, and CD73, as well as their capacities of self-renewal and multilineage differentiation. However, they differ in certain ways. For example, BMMSCs possess greater ability to form colonies and to undergo osteogenic differentiation when compared to LNMSCs. Conversely, LNMSCs show superior capacities for chondrogenic and adipogenic differentiation relative to those of BMMSCs.

T cells are a key regulator of adaptive immune responses. T-cell-mediated immunity proceeds through activation, proliferation, differentiation and cytokine secretion. T-cell activation is required in the initiation of various T lymphocyte functions. After that, the exposure of naive T cells to an antigen leads to their proliferation and differentiation, resulting in the generation of effector T cells and memory T cells. Effector T cells perform their function via the destruction of pathogen and cytokines secretion. At the end of the immune reaction, the apoptosis of the T cells represents the loss of function [18]. LNMSCs empower T cells in multiple respects, including by promoting their proliferation, differentiation, activation and resistance to apoptosis.

Previous studies indicated that MSCs, including BMMSCs and those from adipose tissue, dental pulp and umbilical cord, have the ability to suppress T-cell function by inhibiting their proliferation and secretion of cytokines, as well as inducing T-cell apoptosis [22, 31, 32]. Although it has been observed that MSCs might rescue T cells from apoptosis under starving conditions or other situations that cells physiologically prone to spontaneous cell death [33]. It has also been reported that steroids, together with TNF-α, synergistically induce the function of MSCs to enhance CD8^+^ T-cell response and leads to an exacerbation of GvHD progression [34]. Hence, the underlying mechanism of this bidirectional effects of MSCs remains unclear. In this study, we revealed that LNMSCs are capable of empowering T cells, which is the opposite of the regulatory effect exhibited by other types of MSCs. This feature of LNMSCs mainly presented in our study as the promotion of T-cell activation, proliferation, differentiation, and cytokine secretion, which strongly facilitates the local survival of T cells and boost their function in the LNs. MSCs in lymph organs may play a unique role in immune regulation, and therefore provide a theoretical basis for elucidating the physiological significance of MSCs.

MCP-1 plays a crucial role in immune responses, as it is an important initiator to help T cells leave immune organs and reach the site of local response. *Mcp1* knockout mice show distinct deficiencies in both innate and adaptive immunity. The loss of MCP-1 results in the loss of function and reduction in number of macrophages, neutrophils and leukocytes, and affects the number of Th1 and Th2 cells in the LNs [35, 36]. We found LNMSCs produce and secrete a high level of MCP-1 after co-culture with T cells, through which they promote T-cell proliferation via activation of the Erk pathway. As expected, BMMSCs were able to promote T-cell apoptosis, and this capacity was diminished after treatment with PD-L1 neutralizing antibody. These results are consistent with the previous observation that PD-L1 is important regulator contributing to MSC-based immunosuppression and inducing lymphocyte death via the Akt signaling pathway [27]. However, PD-L1 expression and its upstream NF-κB signaling pathway were inhibited in LNMSCs when co-cultured with T cells, which was closely correlated to the high expression level of MCP-1. Once MCP-1 was repressed, LNMSC-mediated inhibition of PD-L1 was diminished. It has been reported that elevated expression of PD-L1 is accompanied by the elevation of MCP-1 [37], but no direct antagonism or inhibition between them has previously been noted. Furthermore, we demonstrated that MCP-1 affects PD-L1 expression by inhibiting the NF-κB signaling pathway, through which LNMSCs exert an anti-apoptotic effect on T cells. In summary, here we elaborated upon the underlying mechanism by which LNMSCs empower T cells.

In order to further verify the empowering property of LNMSCs, we use CTX-induced immunosuppressed mice to show LNMSCs’ therapeutic effect. CTX is an alkylating agent used in the treatment of solid and hematological malignancies with side effects including immunosuppression. Therefore, CTX is also used as an immunosuppressive agent and to construct immunosuppressed mouse models. In this study, we used CTX-induced immunosuppressed mice to validate the immune empowerment function of LNMSCs. We found that LNMSCs, but not BMMSCs, were able to reverse immune organ atrophy and immune cell death, as well as to promote immune cell viability and proliferation in CTX-induced immunosuppressed mice. This therapeutic effect was associated with LNMSC-mediated secretion of MCP-1.

## Conclusion

Collectively, the studies presented here reveal a previously unrecognized role of LNMSCs in immune-supportive regulation via MCP-1-induced T-cell empowerment. LNMSCs secrete MCP-1 in LNs to promote T-cell proliferation, activation and differentiation, which help T cells to gain the capacity for functional response. Meanwhile, we revealed that MCP-1 inhibits PD-L1 expression via the NF-κB signaling pathway to suppress T-cell apoptosis and thereby enhance T-cell function. This experimental evidence provides new insights into the current understanding of the immunoregulatory effects of tissue-specific MSCs.

## Materials and methods

### Mice

Male C57BL/6 mice were purchased from Sun Yat-sen University, Guangzhou, China, and male *Gli1-Cre*^*ERT2*^ mice and *R26*^*tdTomato*^ mice were purchased from the Jackson Laboratory, USA. We used age-matched 8-week-old male mice from the same background in all animal experiments. Animals were grouped using a method of randomization. No sample size estimation was performed. All these experiments were performed under an institutionally approved protocol (Sun Yat-sen University, SYSU-IACUC-2021-000692).

### Antibodies and reagents

Anti-CD34, CD44, CD45, CD73, Sca-1, CD90, CD105, and 7AAD antibodies were purchased from BD Biosciences. Anti- CD3, CD8, CD4, CD25, FOXP3, IL17, Annexin V, IFN- γ, and IL2 antibodies were purchased from BioLegend. Anti-Runx2, GAPDH, MCP-1, AKT, p-AKT, Erk, p-Erk, NF-κB p65, p-NF-κB p65, p-IKKα/β, and TNF-α antibodies were purchased from Cell Signaling Technology. Anti-ALP, CD3, PD-L1, PD-L2, and NEMO antibodies, were purchased from Abcam. CFSE cell division tracker Kit, anti-β-actin antibody, Lipofectamine™ RNAiMAX transfection reagent, and trypan blue were purchased from Invitrogen. Anti-PPAR-γ, OCN and Fas-L antibodies were purchased from Santa Cruz. Hoechst 33342 was purchased from Sigma. The EDU cell proliferation assay kit was purchased from KeyGEN Biotech. CCK8 Kit was purchased from DOJINDO.

### CTX-induced immunosuppressed mice

Immunosuppressed mice were induced by intraperitoneal injection of CTX (100 mg/kg, Alfa Aesar). A total number of 0.5 × 10^6^ LNMSCs or BMMSCs were infused into immunosuppressed mice intravenously on day 2 after infusion of CTX. All mice were euthanized and analyzed on day 30 after infusion of MSCs. Induced immunosuppressed mice were evaluated as previously described [38]. No blind method is involved for the animal study.

### Dextran sulfate sodium (DSS)-induced chronic colitis

DSS-induced experimental chronic colitis has been widely used as an inflammatory model, and these mice show symptoms including inflammatory swollen lymph nodes. C57BL/6 mice were supplied with 1.5% DSS in their drinking water for 1 month. On day 30, these chronic colitis mice were sacrificed and their lymph nodes were isolated.

### Isolation and culture of LNMSCs and BMMSCs

Stem cells from mouse bone marrow and LNs were isolated from 8-week-old male mice and cultured as reported previously [25]. We further identified these MSCs and demonstrated that LNMSCs and BMMSCs showed the capacity of self-renewal, assessed by EdU staining assay and colony-forming units. Flow cytometric analysis confirmed the expression of MSC surface markers. The osteogenic, adipogenic and chondrogenic differentiation induction and assessment of LNMSCs and BMMSCs were conducted as reported previously [25].

### Isolation of mouse T cells

Lymphocytes were prepared by disruption of the spleen through a 40-μm mesh and single-cell suspensions were prepared using red blood cell lysis (Cwbio). CD3^+^ T cells were enriched by magnetic cell sorting using the mouse MojoSort CD3^+^ T-cell selection kit (Biolegend) according to the manufacturer’s instructions, CD4^+^ T cells were enriched by magnetic cell sorting using the mouse CD4^+^ T-cell IMagTM particles (BD) in accordance with the manufacturer’s instructions.

### In vitro immunomodulatory capacity of LNMSCs and BMMSCs

T cells, pre-stimulated by anti-CD3 and CD28 antibodies (Biolegend) for 24 h, were directly loaded onto LNMSCs and BMMSCs (5 × 10^5^/per well). T cells and MSCs were co-cultured for 1 day for T-cell apoptotic rate detection, 2 days for T-cell activation and differentiation detection and 1–4 days for T-cell proliferation rate detection. T cells used in activation detection were not pre-stimulated. The number of T cells was detected by flow cytometry. The cytokine array analysis (Mouse Cytokine Array Panel A, R&D Systems) was performed using the cell culture supernatants collected after co-cultured for 1 day in accordance with the manufacturer’s instructions.

### Flow cytometry analysis

For cell surface marker analysis, we prepared cells in 100 μl suspension at a density of 10^6^ cell. Then we added 1 μl antibody into the suspension and incubated for 30 min on ice. The apoptotic T cells were stained with CD3 antibody at 4 °C for 30 min and washed, followed by incubation of Annexin V and 7AAD antibody. The flow cytometry analysis was performed using flow cytometry (ACEA NovoCyteTM).

### Western blotting

Cells or tissue were lysed in protein extraction reagent (Thermo Fisher Scientific) with phosphatase and protease inhibitors (Roche). The samples were quantified by protein concentration assay (Bio-Rad Laboratories). 20 µg of proteins were separated by SDS-polyacrylamide precast gels (Invitrogen) and transferred to nitrocellulose membranes (Millipore). The membranes were blocked with 5% bovine serum albumin and 0.1% Tween 20 for 1 h. The membranes were incubated with primary antibodies overnight at 4 °C, and then incubated at room temperature for 1 h in secondary antibody diluted at 1:1000 in blocking buffer.

### siRNA knockdown, plasmid transfection, and cytokine treatments

For siRNA knockdown, MCP-1 siRNA (OBiO Technology) was used to treat the LNMSCs (2 × 10^5^) in accordance with the manufacturer’s instructions. Nontargeting control siRNAs (OBiO Technology) were used as negative controls. PDL1-EGFP protein overexpression plasmids (OBiO Technology) were used. Moreover, we used empty plasmids with the same backbone as a control. The LNMSCs were transfected with plasmids using Lipofectamine 3000 (Life Technologies). The efficiency of siRNA knockdown and plasmid transfection were confirmed by Western blot analysis. After transfection, LNMSCs were co-cultured with pre-activated T cells for 1 day. For cytokine treatments, I-LNMSCs and BMSCs were treated with MCP-1 (Peprotech) for 48 h, and meanwhile co-cultured with pre-activated T cells. After cytokine treatment or transfection, MSCs protein were extracted for Western blotting, T cells were collected for flow analysis and the culture supernatants were used for ELISA.

### Immunofluorescence staining

To detect the in vivo distribution of MSCs in LNs and bone marrow, *Gli1-Cre*^*ERT2*^ mice were crossed with *R26*^*tdTomato*^ mice. Tamoxifen (Sigma) was infused into *Gli1-Cre*^*ERT2*^*; R26*^*tdTomato*^ mice intraperitoneally. At 48 h post-infusion, the LNs and femurs were collected and fixed with 4% PFA, and then femurs were decalcified with 5% EDTA for 1 week. Samples were embedded in OCT compound (Sakura Finetek). Frozen sections were performed and the slides were stained with CD3 antibody, followed by secondary antibody staining. Slides were mounted with mounting medium containing DAPI (Abcam).

For detection of in vivo T-cell proliferation and apoptosis, EdU (Ribo Bio) was infused into CTX-induced mice intra-peritonelly 24 h before sacrifice. Frozen sections of spleens were prepared, then kFluor488-EDU cell proliferation assay kit was used to stain the proliferated cells and DeadEnd™ Fluorometric TUNEL System Kit (Promega) was used to stain the apoptotic cells, slides were then stained with CD3 antibody, followed by secondary antibody staining. Finally, slides were mounted with mounting medium containing DAPI.

### Enzyme-linked Immunosorbent (ELISA) Assay

TNF-α, IFN-γ and IL-6 were measured with ELISA kits purchased from R&D Systems, and MCP-1 were measured with ELISA kit purchased from Abcam, according to the manufacturers’ instructions.

### Real-time quantitative polymerase chain reaction (RT-qPCR)

Total RNA was extracted from the cells and tissues using the miRNeasy Mini Kit (Qiagen) in accordance with the manufacturer’s instructions. The RT-qPCR was performed using SuperScript III (Life Technologies), SYBR Green Supermix (Bio-Rad) and gene-specific primers (Table 2), and was detected on a CFX96™ Real-Time PCR System (Bio-Rad).

**Table 2:** The primers used in the RT-qPCR assay.

Primers	Forward	Reverse
MCP-1	5′-GTCCCTGTCATGCTTCTGG-3′	5′-GCTCTCCAGCCTACTCATTG-3′
JAK-1	5′-TGAGGGACATTAACAAGCTGG-3′	5′-AGCTCAACCTTCCCAAAGTG-3′
PD-L1	5′-CTCATTGTAGTGTCCACGGTC-3′	5′-ACGATCAGAGGGTTCAACAC-3′
GAPDH	5′-CACCATGGAGAAGGCCGGGG-3′	5′-GACGGACACATTGGGGGTAG-3′

### iTRAQ analysis

T cells were isolated using a mouse MojoSort CD3^+^ T-cell selection kit and pre-stimulated with anti-CD3 and CD28 antibodies for 24 h as described above. Subsequently 5 × 10^6^ T cells were loaded onto cultured LNMSCs and BMMSCs (1 × 10^6^/per well). After LNMSCs and BMMSCs were co-cultured with T cells for 1 day, cells in the suspension were removed and the adherent cells were collected. The Mouse MojoSort CD3^+^ T-cell selection kit was used to enrich CD3-negative MSCs from the adherent cells.

After co-cultured with T cells for 1 day, total LNMSC or BMSC proteins (100 μg) were lysed in protein extraction reagent (Thermo Fisher Scientific) with phosphatase and protease inhibitors (Roche). The samples were quantified by protein concentration assay (Bio-Rad Laboratories). Afterwards, the proteolysis and iTRAQ labeling of the peptides were conducted. The Shimadzu LC-20AB liquid phase system was used to fractionate peptides. The samples were combined according to the chromatographic elution peak map. Separation was performed by using Thermo UltiMate 3000 UHPLC. The peptides separated by liquid phase chromatography were ionized by a nanoESI source and then passed to a tandem mass spectrometer Q-Exactive HF X (Thermo Fisher Scientific, San Jose, CA) for DDA (Data Dependent Acquisition) mode analysis.

The raw MS/MS data were converted into MGF format and the exported MGF files were searched by the local Mascot server comparing the database. In addition, protein quantification was performed using IQuant with quality control. All proteins with a false-discovery rate (FDR) <1% were proceed with GO analysis. Further, deep analysis based on differentially expressed proteins, including Gene Ontology (GO) enrichment analysis and KEGG pathway enrichment analysis, were performed.

### Statistical analysis

SPSS 20.0.0 was used to perform statistical analysis. According to the homogeneity test of variance, comparisons between two groups were analyzed using independent two-tailed Student’s *t*-tests, while comparisons between more than two groups were analyzed using one-way ANOVA. *P*-values < 0.05 were considered statistically significant.

## Supplementary information

Reproducibility checklist

Supplementary Figures

Original Western blots

## Data Availability

All data needed to evaluate the conclusions in this study are presented in the paper. Additional data related to this paper may be requested from the corresponding authors.
