# Supplementary material for: Mesenchymal stem cells empower T cells in the lymph nodes via MCP-1/PD-L1 axis
Source: Cell Death Dis. 2022 Apr 18;13(4):365. doi: 10.1038/s41419-022-04822-9 (PMC9016066; doi:10.1038/s41419-022-04822-9)
Supplement: Supplementary file 2 — Supplementary Figures [file 41419_2022_4822_MOESM2_ESM.docx]

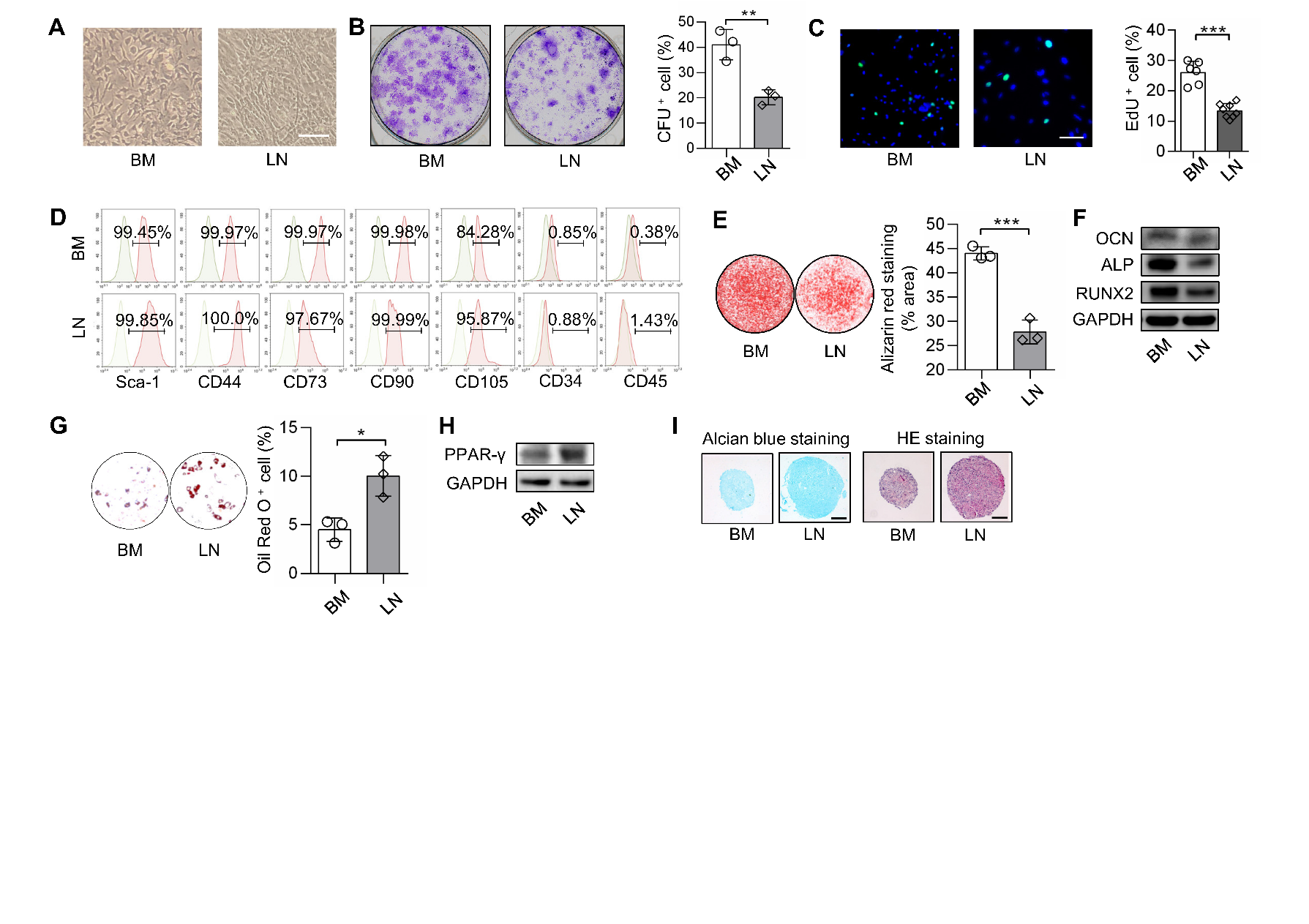
**Figure S1. Characterization of LNMSCs.** (A) Representative cell morphology of LNMSCs and BMMSCs. Scale bar = 100 μm. (B-C) Proliferation rate and colony-forming capacities of LNMSCs and BMMSCs. Scale bar = 100 μm. n =3 (B), n = 6-8 (C). (D) Flow cytometry analysis of LNMSCs and BMMSCs. LNMSCs were negative for CD34 and CD45, positive for Sca-1, CD44, CD73, CD90 and CD105. (E-I) Alizarin red staining, oil red O staining and alcian blue-staining showed the osteogenic, adipogenic and chondrogenic differentiation of LNMSCs and BMMSCs. Western blot analysis confirmed the expression of the osteoblastic markers OCN, ALP and RUNX2; adipogenic markers PPAR-γ. GAPDH was used to assess the amount of protein loaded per sample. Scale bar = 200μm. n = 3. *: *P* < 0.05, **: *P* < 0.01, ***: *P* < 0.001.


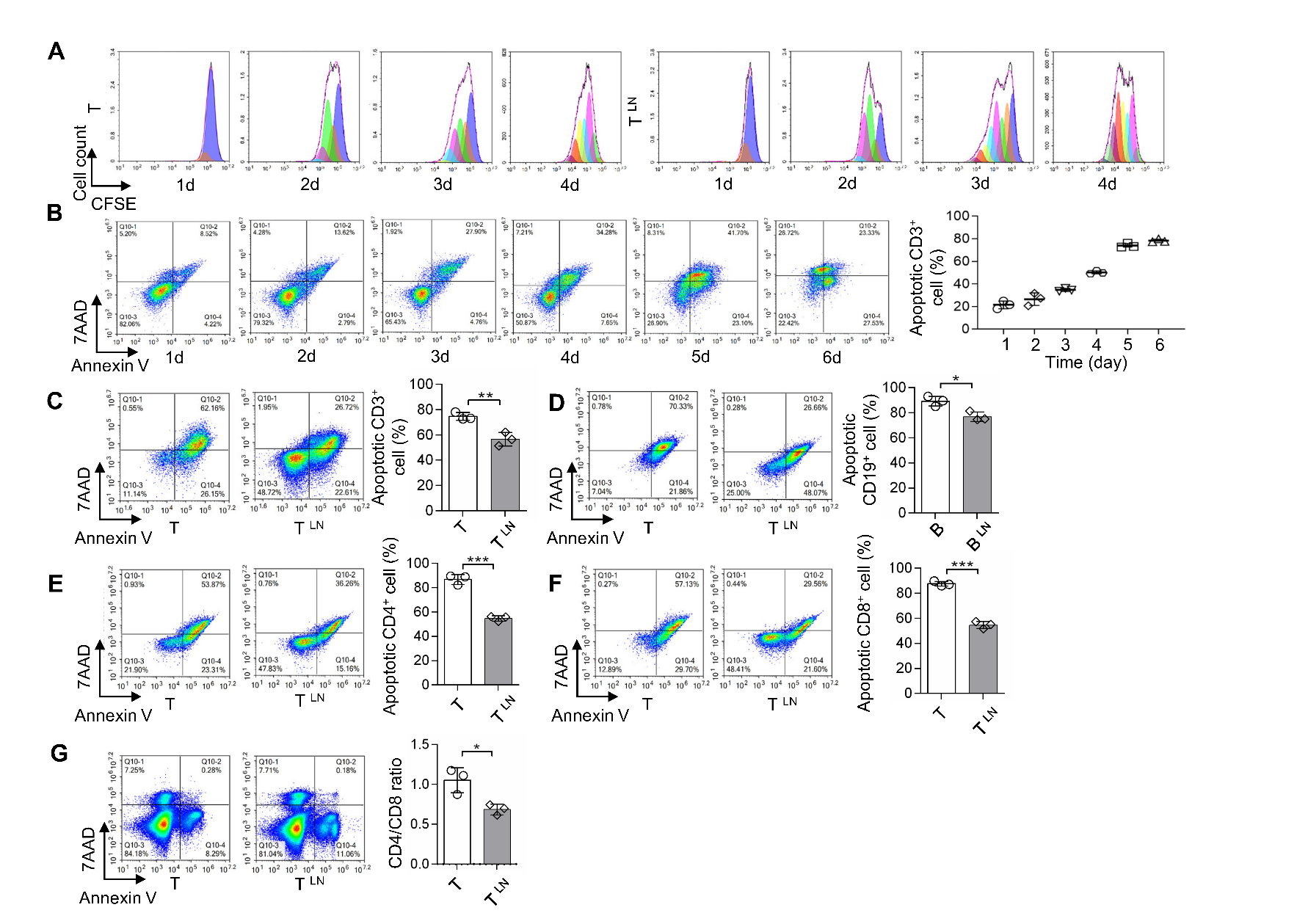
**Figure S2. LNMSCs regulate lymphocytes *in vitro*.** (A) CFSE analysis showed that LNMSCs promote the proliferation of activated T cells from day 1-4. (B) Flow cytometry analysis showed that the rate of T cell apoptosis increased significantly on days 3-4 after activation. n = 3. (C) LNMSCs supported the survival of activated T cells on day 5 of the co-culture. n = 3. (D-F) LNMSCs supported the survival of activated CD19^+^ B cells, CD4^+^ and CD8^+^ T cells on day 5 of co-culture. (G) LNMSCs affected the ratio of CD4^+^/CD8^+^ T cells on day 5 of co-culture. n = 3. *: *P* < 0.05, **: *P* < 0.01, ***: *P* < 0.001.


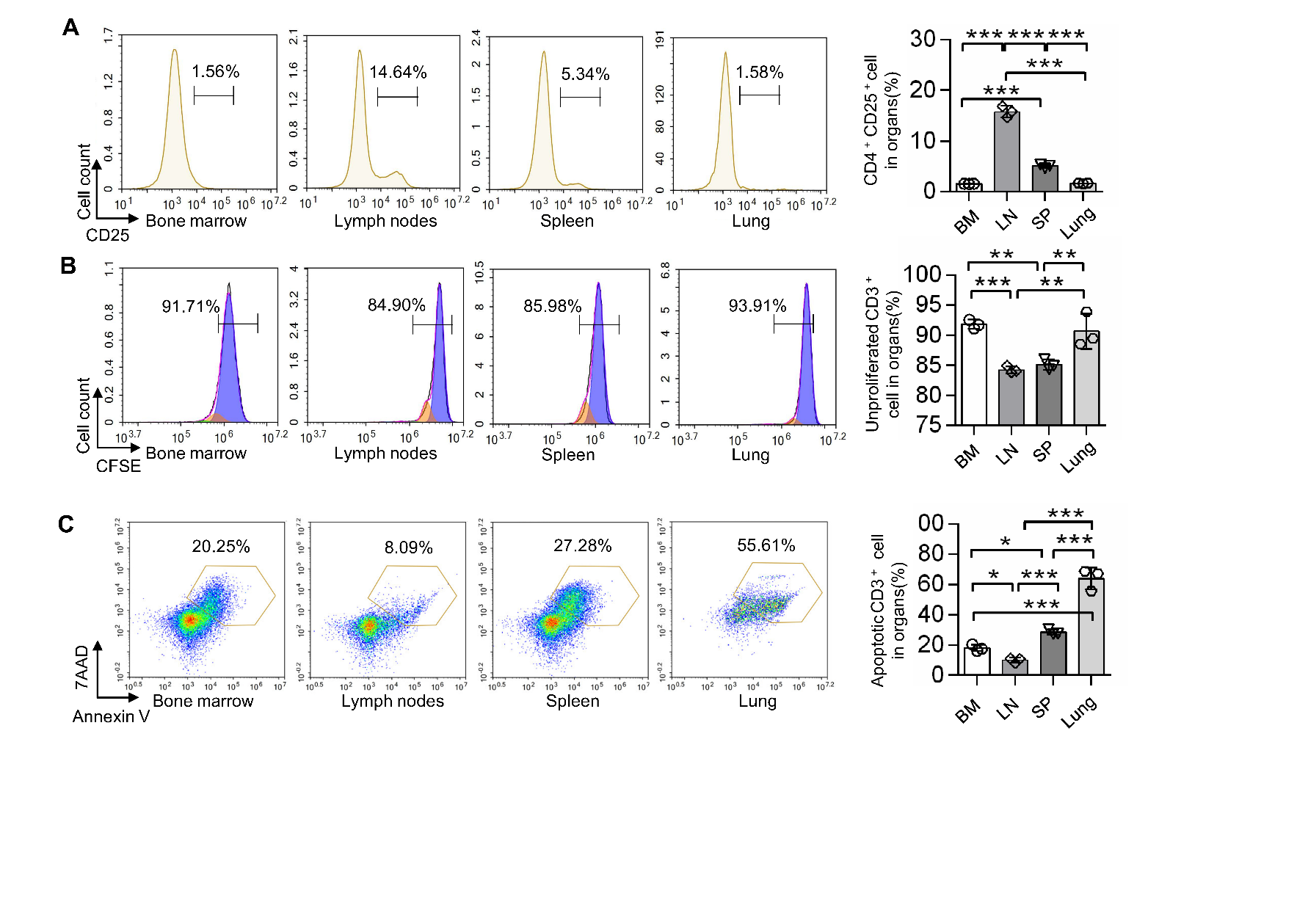
**Figure S3. T cell function is activated in lymph nodes.** (A) Activation rate of T cells of different organs including the bone marrow, lymph nodes, spleens and lungs. n = 3. (B) Proliferation rate of T cells derived from different organs including the bone marrow, lymph nodes, spleens and lungs. n = 3. (C) Apoptotic rate of T cells of different organs including the bone marrow, lymph nodes, spleens and lungs. n = 3. *: *P* < 0.05, **: *P* < 0.01, ***: *P* < 0.001.


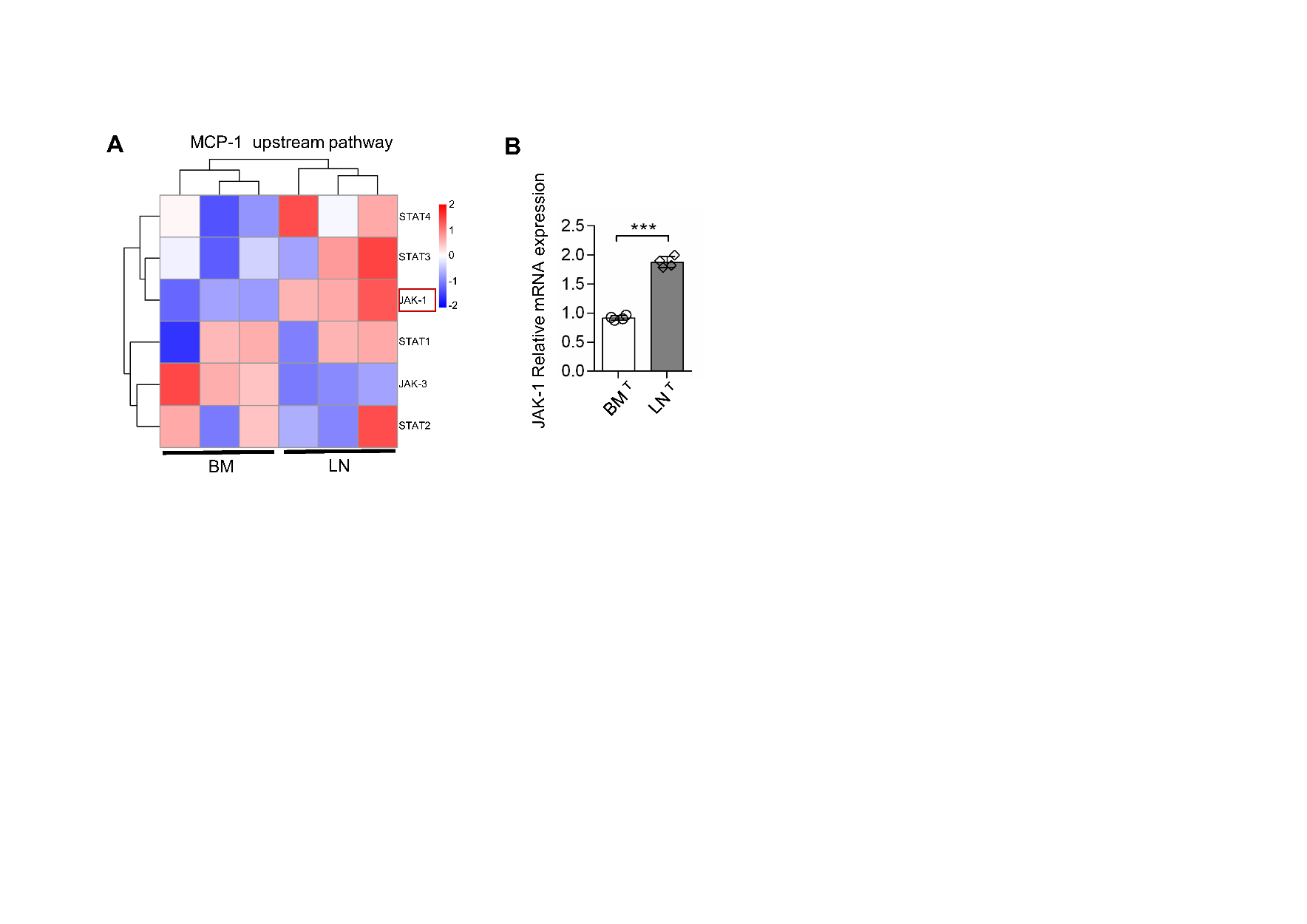
**Figure S4. LNMSCs express a higher level of JAK-1 than BMMSCs.** (A) iTRAQ quantification proteomic results showed LNMSCs express higher levels of JAK-1, STAT3 and STAT4 than BMMSCs. n = 3. (B) RT-qPCR confirmed that LNMSCs express a higher level of JAK-1 than BMMSCs. n = 4. ***: *P* < 0.001.


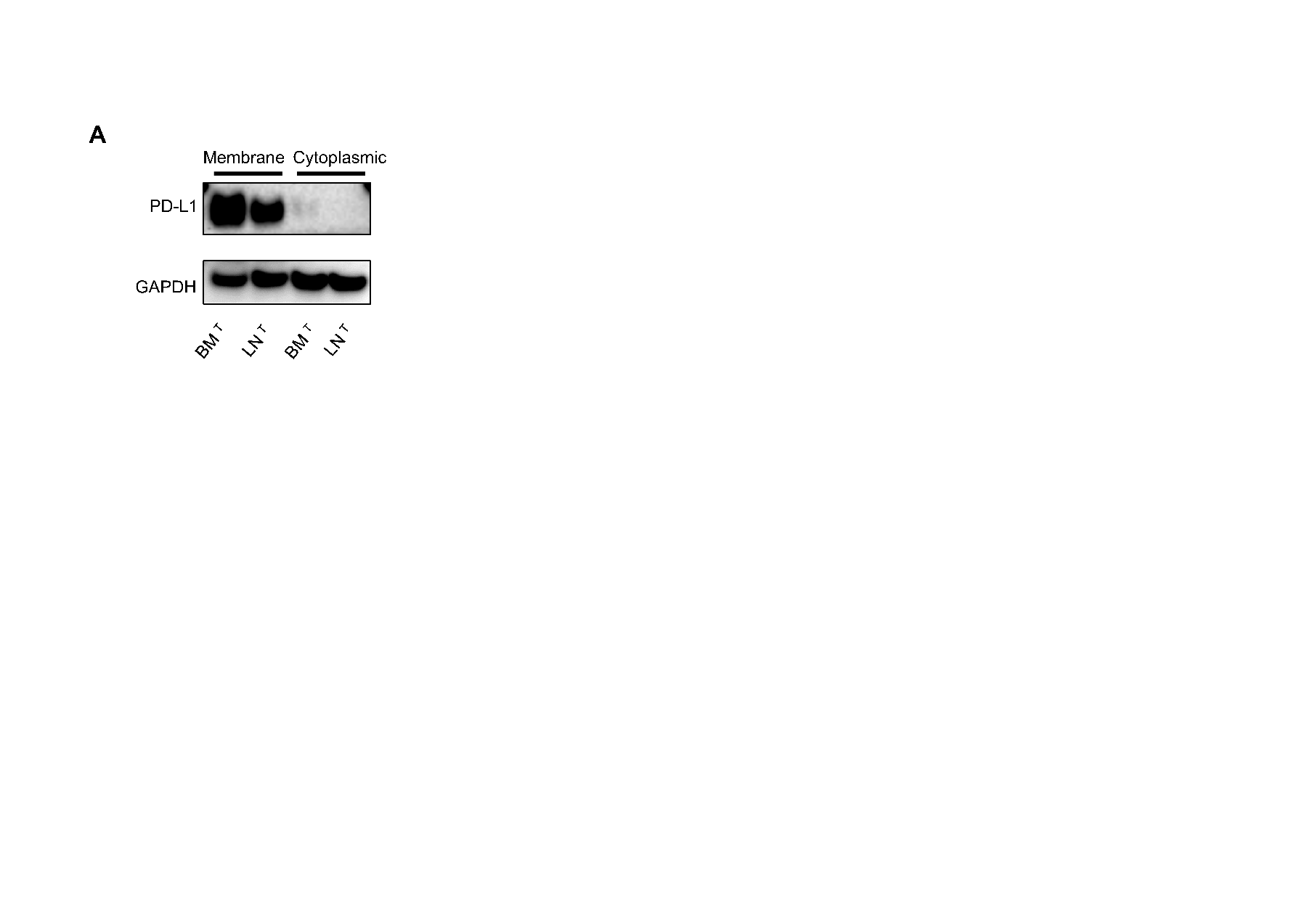
**Figure S5. LNMSCs express PD-L1 in the cytomembrane.** (A) Both LNMSCs and BMMSCs express PD-L1 mainly in the cytomembrane.


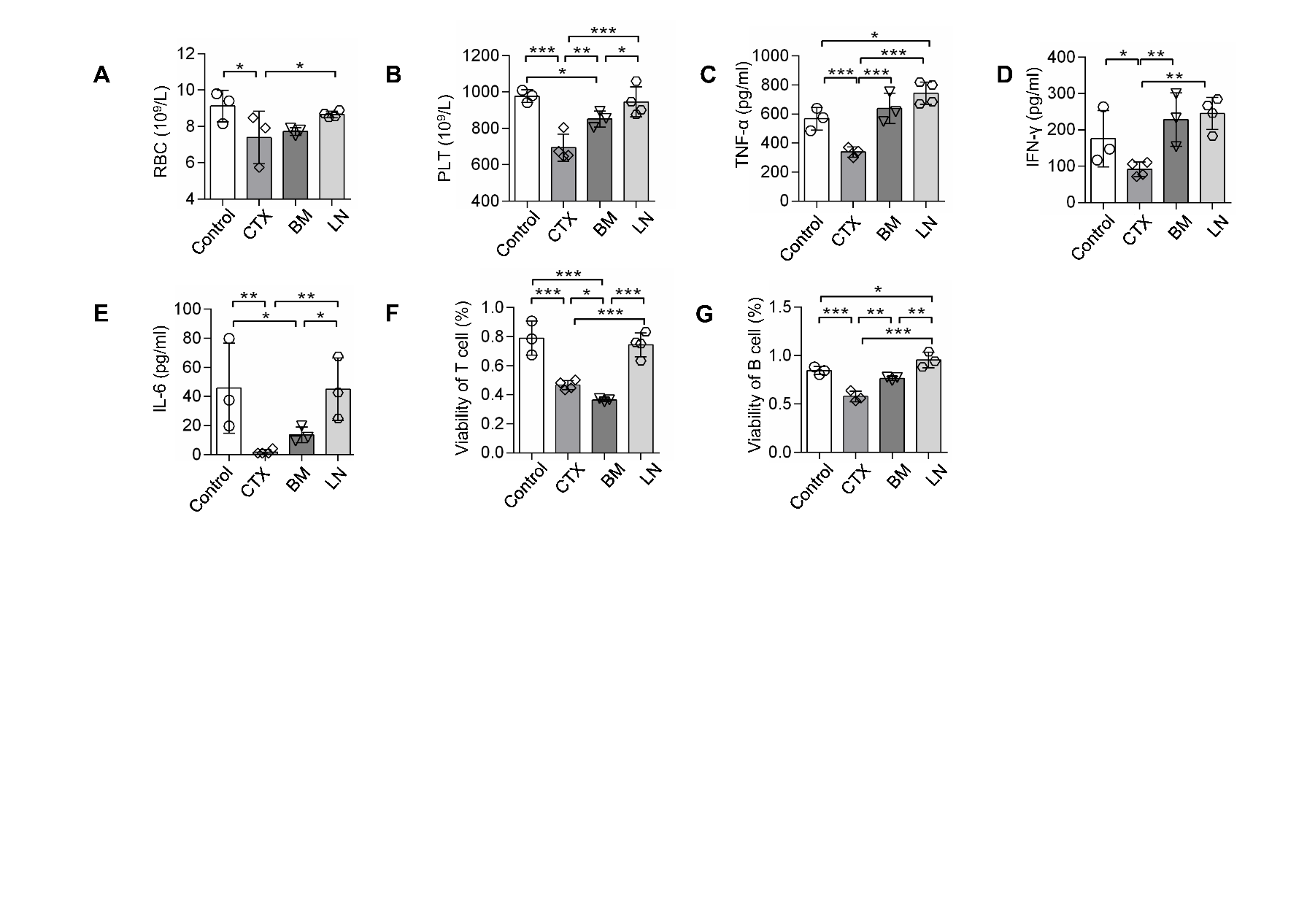
**Figure S6. LNMSCs have immunosupportive qualities in CTX-induced immunosuppressed mice.** (A-B) The RBC, PLT in the blood of CTX-induced immunosuppressed mice. n = 3-4 (A), n = 3-4 (B). (C-E) ELISA analysis showed the levels of TNF-α, IFN-γ and IL-6 in the blood of CTX-induced immunosuppressed mice. LNMSC treatment improved the reduced levels of TNF-α, IFN-γ and IL-6, while BMMSC treatment only improved the levels of TNF-α and IFN-γ. n = 3-4 (C-E). (F-G) CCK8 assay showed the viability of T cells and B cells of lymph nodes recovered after treatment with LNMSCs. n = 3-4 (F), n = 3 (G). *: *P* < 0.05, **: *P* < 0.01, ***: *P* < 0.001.


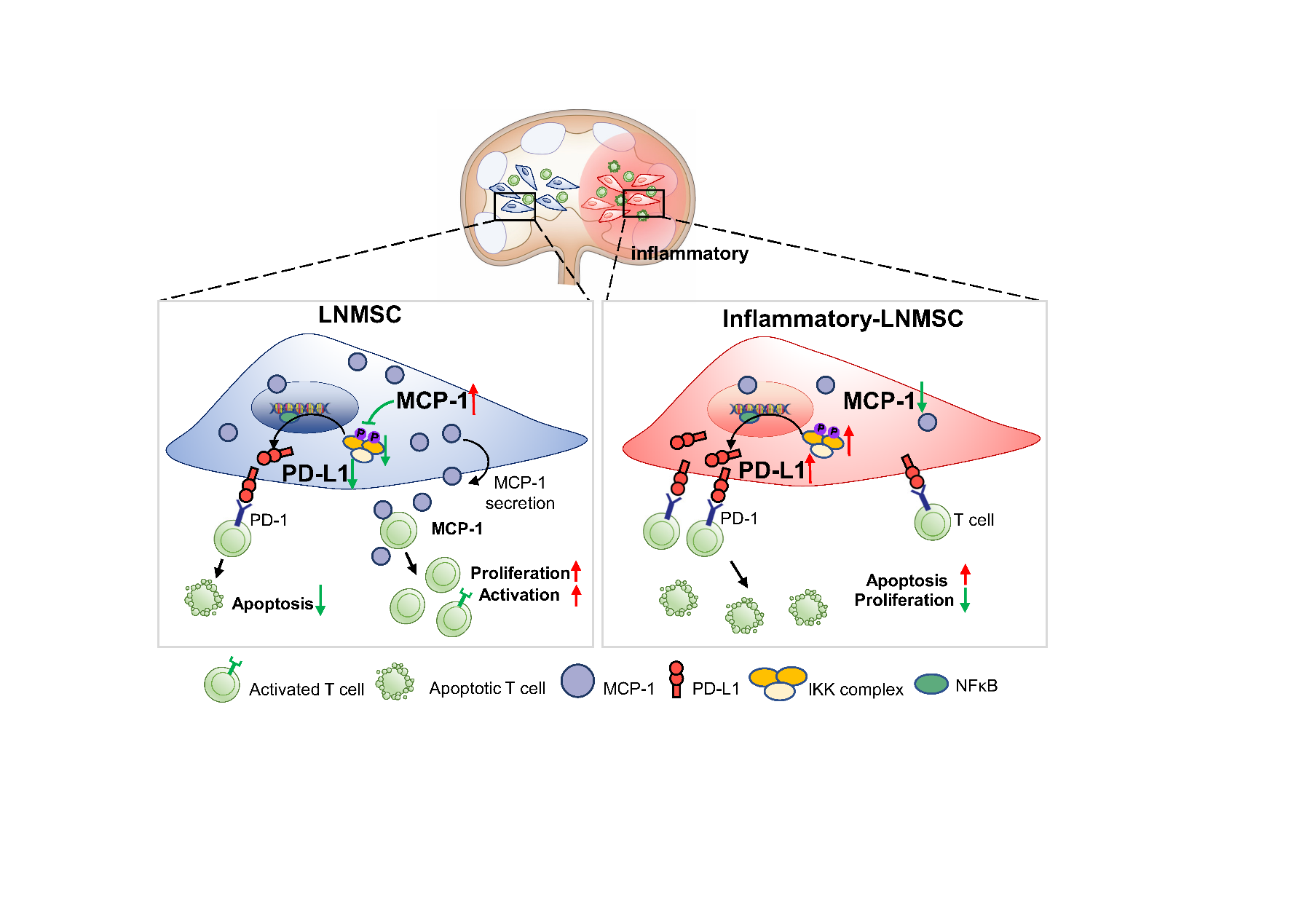
**F****igure S7. LNMSCs secrete MCP-1 to promote T cell proliferation and suppress T cell apoptosis *via* MCP-1/PDL1 axis.**
