# Supplementary figures and images for: Mesenchymal stem cells empower T cells in the lymph nodes via MCP-1/PD-L1 axis
Source: Cell Death Dis. 2022 Apr 18;13(4):365. doi: 10.1038/s41419-022-04822-9 (PMC9016066; doi:10.1038/s41419-022-04822-9)

# Original Western blots

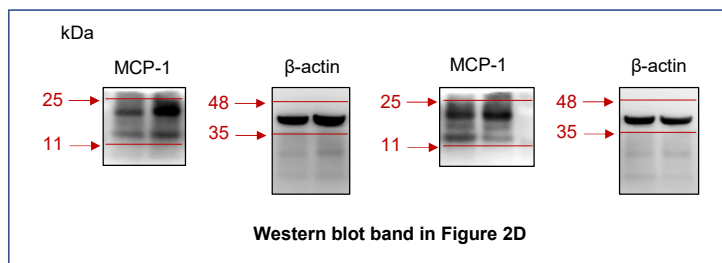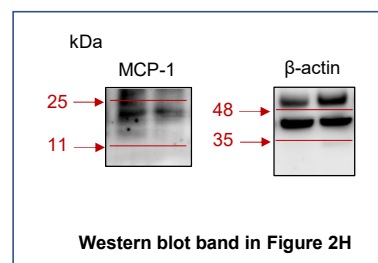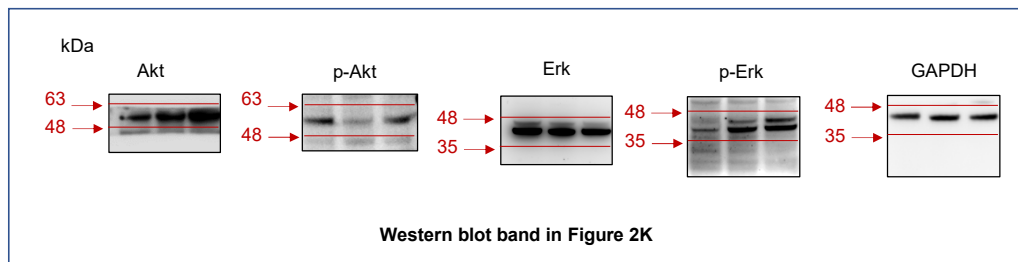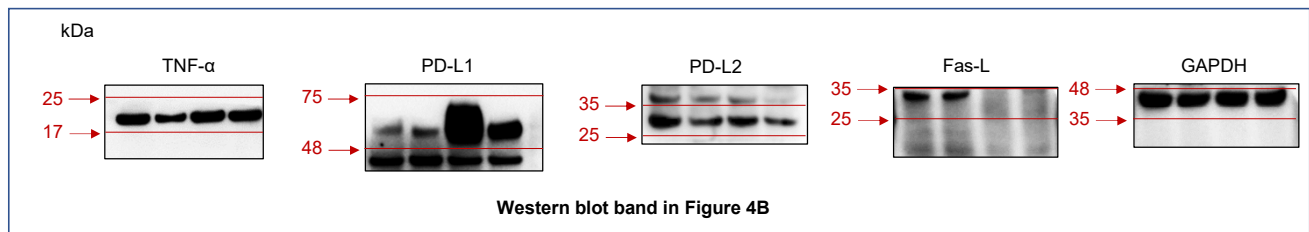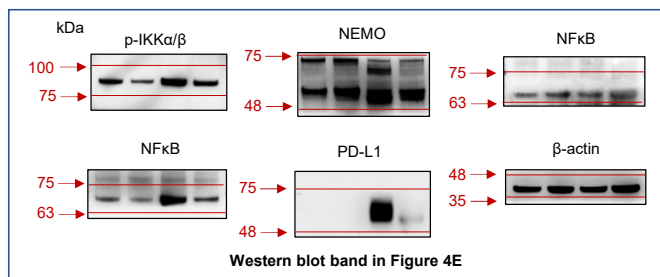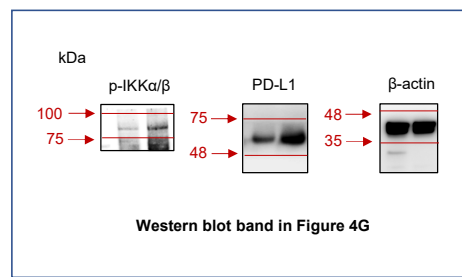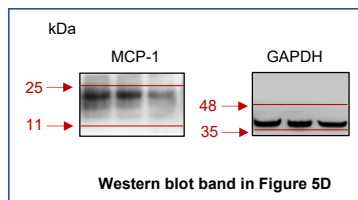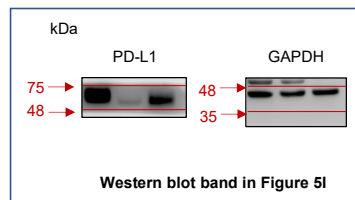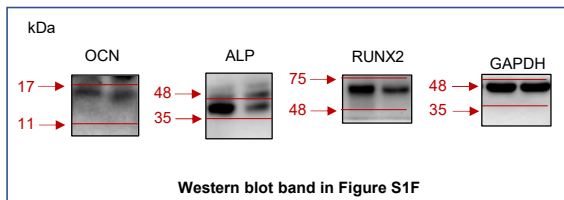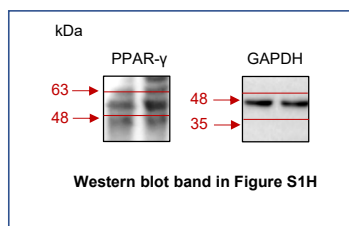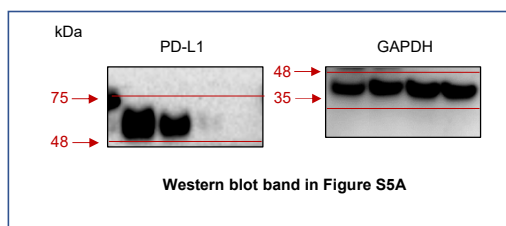

Supplement: Supplementary file 3 — Original Western blots [file 41419_2022_4822_MOESM3_ESM.pdf]
